# The Effects of *VEGF-A* and *GSTM1*/*GSTT1* Variants in the Susceptibility to the Chronic Rhinosinusitis with Nasal Polyposis: A Pilot Genetic Study

**DOI:** 10.3390/biomedicines12102383

**Published:** 2024-10-18

**Authors:** Leandro Azevedo Camargo, Angela Adamski da Silva Reis, Stela Oliveira Rodrigues, Rodrigo da Silva Santos, Melissa Ameloti Gomes Avelino

**Affiliations:** 1 Departamento de Cirurgia, Faculdade de Medicina, Universidade Federal de Goiás, Goiânia 74.690-900, GO, Brazil; leandroazevedocamargo@gmail.com (L.A.C.); stelarodrigues@outlook.com (S.O.R.); melissaameloti@ufg.br (M.A.G.A.); 2 Departamento de Bioquímica e Biologia Molecular, Instituto de Ciências Biológicas, Universidade Federal de Goiás, Goiânia 74.690-900, GO, Brazil; rdssantos@ufg.br

**Keywords:** vascular permeability, inflammation, oxidative stress, Brazilian population

## Abstract

Nasal polyps (NPs) are usually part of chronic rhinosinusitis with nasal polyposis (CRSwNP). However, the exact etiology of CRSwNP is still unknown. In addition, the suggested etiological causes are infection, allergy, and immunological disorders, among others, such as genetic predisposition. Moreover, it is also suggested that oxygen-free radicals play a vital role in the pathogenesis of nasal polyposis, and inflammatory cells produce free radicals during phagocytosis, which is the primary source of ROS, controlled by the glutathione S-transferase (GST) system. Although, vascular endothelial growth factor (VEGF) plays an important role in angiogenesis, it is closely interwoven with the mobilization of inflammatory cells. This pilot study evaluated the association between genetic variant *VEGF-A* (rs28357093) and *GSTM1*/*GSTT1* deletion polymorphism in susceptibility to CRSwNP. A case–control study was conducted with 61 individuals diagnosed with CRSwNP and 100 healthy subjects. *VEGF-A* (rs28357093) and *GSTM1*/*GSTT1* deletion polymorphisms were genotyped by RFLP-PCR and SYBR Green real-time PCR, respectively. Individuals with allergic rhinitis carriers with AC genotype (rs28357093) presented a 4-fold increased risk to CRSwNP (OR = 4.20, 95% CI = 1.31 to 13.50; *p* = 0.015). This evidence shows that the increased vascular permeability probably causes an inflamed nasal area leading to extensive edema and polyp growth. On the other hand, no association was verified for each genetic variant by inheritance models. Interestingly, the *GSTT1* present genotype showed a protective effect on CRSwNP. In conclusion, additional studies that have larger groups in different geographic localizations may be useful to verify and assess the association between genetic variants and CRSwNP.

## 1. Introduction

Nasal polyps (NPs) are usually part of chronic rhinosinusitis with nasal polyposis (CRSwNP). This pathology is characterized by inflammation in the nose and paranasal sinus [1]. The diagnostic criteria for CRSwNP include two or more symptoms, one of which should be either nasal blockage/obstruction/congestion or nasal discharge, associated with facial pain/pressure and/or a reduction in smell that lasts for ≥12 weeks in the presence of polyps in the nasal cavity [2,3]. However, the exact etiology of CRSwNP is still unknown. There are several viewpoints for identifying the etiology of this pathology. Moreover, the suggested etiological causes are infection, allergy, and immunological disorders, among others [4,5].

An interesting mediator with a putative role in CRSwNP formation is vascular endothelial growth factor (VEGF) [6]. VEGF is involved in angiogenesis; it increases vascular permeability and is expressed in inflamed nasal and middle ear mucosa. In most situations, if not all, angiogenesis is closely interwoven with the mobilization of inflammatory cells [6]. During physiological or repair processes, such as wound healing, the inflammation process is transient; most pathological conditions involve a continuous recruitment of inflammatory cells, which, in turn, serve as a substantial source of reactive oxygen species (ROS) [4].

Therefore, it is suggested that oxygen-free radicals play a vital role in the pathogenesis of nasal polyposis, and inflammatory cells produce free radicals during phagocytosis, which is the primary source of ROS [7,8,9]. These free radicals are implicated in the development of numerous diseases and inflammatory conditions. ROS and oxidative stress are suggested as possible factors in the development of nasal polyps, and there are two protective mechanisms (enzymatic and nonenzymatic) to prevent tissue damage [8,9].

Additionally, there is a functional connection between the inflammation-dependent generation of ROS, and angiogenesis plays an important role during various stages of initiation to vascularization [6]. Under inflammatory conditions, oxidative stress produced by polymorphonuclear neutrophils (PMNs) leads to the opening of inter-endothelial junctions and promotes the migration of inflammatory cells across the endothelial barrier. Glutathione S-transferase (GST) is an important enzyme family that is involved in the elimination of ROS contributing to detoxification and cell protection and genetic polymorphisms have been involved in predisposing to some inflammatory diseases [10,11,12,13].

Genetic predisposition to CRwNP has been suggested by some studies. Genome-wide association studies (GWAS) have identified thousands of genetic variants linked to the risk of human disease [8]. For a better understanding of oxidative stress-mediated mechanisms involved in inflammation and tissue injury, genetic variants are proposed to predict the risk of CRSwNP. Thus, the current study evaluated the association between the variants *VEGF-A* -141 A > C (rs28357093) and *GSTM1*/*GSTT1* deletion polymorphism in susceptibility to CRSwNP.

## 2. Materials and Methods

### 2.1. Subjects

A total of 61 individuals diagnosed with CRSwNP (case group) and 100 healthy subjects (control group) were included in this study. The nasal polyposis diagnosis was made by a clinical examination consisting of anterior rhinoscopy, nasal endoscopy with a flexible telescope, and paranasal sinus computed tomography. The patients were not using any nasal or systemic corticosteroids or antibiotics eight weeks before the biopsy. The study was performed at Hospital das Clínicas—Faculdade de Medicina from Universidade Federal de Goiás (UFG), Brazil. The control group consisted of individuals selected from the general population. Both groups were matched by sex and age, eliminating the possibility of a confounding factor. The inclusion criteria were established according to The Strengthening the Reporting of Genetic Association Studies (STREGA) guidelines for improved reporting of genetic association studies [14].

Data on life habits, such as smoking, general health conditions, family history, and other medical history information were collected during the interviews. Patients who reported smoking for more than one year before diagnosis were considered smokers. Moreover, this study was conducted according to the Research Ethics Committee (84945720.8.0000.5078) of the UFG and followed the Ethical Principles for Medical Research Involving Human Beings of the Declaration of the World Medical Association of Helsinki. All participants provided written informed consent.

### 2.2. Genotyping and Statistical Analysis

For molecular analysis, peripheral blood DNA was extracted using PureLink™ (Thermo Fisher Scientific, Waltham, MA, USA). Genomic DNA (Invitrogen^®^, Waltham, MA, USA) and genotypes were determined by Polymerase Chain Reaction—Restriction Fragment Length Polymorphism (PCR-RFLP). The PCR amplicons for the *VEGF-A* gene (rs28357093) were digested with *HhaI* restriction enzyme and was performed in 15% polyacrylamide gel electrophoresis (PAGE), stained with silver nitrate. The detailed protocol is under the DOI number: https://doi.org/10.17504/protocols.io.bqvymw7w, accessed on 20 June 2024.

*GSTM1* and *GSTT1* deletion polymorphisms were determined by Multiplex Real-Time Polymerase Chain Reaction using the fluorophore SYBR^®^ Green I (Sso Advanced™ Universal SYBR^®^ Green Supermix- Bio-Rad, Hercules, CA, USA), with discrimination of the null and present genotypes by analysis of the melting curves generated after the amplification reactions. Co-amplification of the *RH92600* gene, a microsatellite region, was performed for endogenous control of the reaction. The details of the protocol are available at https://doi.org/10.17504/protocols.io.bvvvn666, accessed on 20 June 2024.

Statistical analysis was performed using the RStudio^®^ (version 4.0.2) and the SNPStats^©^ software (available at https://www.snpstats.net/start.htm). The general characteristics of the study population were compared by Student’s t and Fisher’s exact tests. Association of the single nucleotide variants (SNVs) and deletion polymorphism with CRSwNP were assessed by logistic regression, and allelic association was evaluated by Pearson’s chi-squared test. For all tests, the significance level was set at 5%.

## 3. Results

Table 1 shows the demographic characteristics of this pilot study. The variable age showed a significant association with the disease (*p* > 0.05). However, smoking was higher among the control group (*p* = 0.00000010). We observed a male predominance in the case group.

For genetic association analysis, the findings of genotypic frequencies for the different inheritance models (codominant, dominant, recessive, and over dominant) in the patients with CRSwNP and controls are presented in Table 2. Furthermore, no evident risk for developing the disease was observed in the inheritance models evaluated for the *VEGF-A* gene—rs28357093 (Table 2). Thus, these findings showed that no association was statistically significant.

For *GST* deletion polymorphism, the null genotype is defined as the complete deletion of both alleles (homozygous deletion), and the presence of at least one allele characterizes the present genotype. In the dominant model, there was a reduced risk for the *GSTM1* null genotype assessed (OR = 0.50; 95% CI = 0.240–1.05; *p* = 0.06), with a significant trend. Additionally, the findings described a non-association between *GSTT1* deletion polymorphism with CRSwNP risk (OR = 0.51; 95%CI = 0.26–0.99, *p* = 0.05). Interestingly, these data could describe that the *GSTT1* present genotype favors a protective effect in the control of oxidation stress (Table 2).

Furthermore, the genetic association analysis did not allow for inferring risk in susceptibility to disease in the inheritance models. In this context, it was not necessary to investigate whether the combination of genotypes (*GSTM1*/*GSTT1* and *VEGF-A* genes) could be associated with the CRSwNP’s risk due to the reduced risk presented in the analysis in the inheritance models (Table 2).

As shown in Table 3, the risk association of *VEGF-A* and *GSTM1*/*GSTT1* genotypes were not significantly associated with asthma and surgery, respectively. On the other hand, for allergic rhinitis, there was a 4-fold increased CRSwNP risk in the individuals’ carriers of the AC genotype (OR = 4.20, 95% CI = 1.31 to 13.50; *p* = 0.015). The presence of the C allele in the heterozygous genotype conferred a risk.

## 4. Discussion

In this pilot study, we evaluated genetic variants in a group of patients’ CRSwNP, as well as in matched controls, both selected from a Brazilian region. There is an ongoing dilemma concerning the pathogenesis of CRSwNP as few studies have been published regarding association between genetic variants and disease risk [3,8]. Our results showed that the study group had an average age of 57.7 years, and there was a male prevalence (50.8%). These findings corroborate with other epidemiological studies [14]. On the other hand, smoking does not appear to be a strong risk factor for CRSwNP [4].

The results obtained in this genetic epidemiologic study indicated that *VEGF-A* and *GSTT1*/*GSTT1* polymorphisms were not associated with the risk of CRSwNP development. However, the AC genotype (*VEGF-A* gene) conferred an important susceptibility to CRSwNP in allergic rhinitis patients. Besides that, a contribution of the C allele was also verified with an increased risk to developing the disease in all inheritance models for the rs28357093, but these findings were not statistically significant.

For genetic inheritance models, there was no risk association between the genetic variants and CRwNP. These findings may be explained by the multifactorial disease character that may result in the interactions of genes, environmental exposure, lifestyle, infection, allergy, and immunological disorders. Thus, CRwNP is a complex chronic disease displaying contributions from multiple genetic and environmental factors. Considering other studies carried out in published studies, there is evidence for the implication of genetic factors in the pathophysiology of disease, however with inconsistent conclusions [8,15]. Thus, advances in technology could facilitate genetic studies in CRSwNP. Additionally, genetic variants have been considered important in the definition of the susceptibility profile for the development of this disease, and our study describes the first findings concerning *VEGF-A* (rs28357093) and *GSTM1*/*GSTT1* variants in the Brazilian population.

Genetic variants in the promoter or exon region might affect gene expression or protein functions and influence different characteristics among individuals. Functional genetic polymorphisms which alter the regulation of gene expression are anticipated to have a substantial influence on disease pathogenesis [16]. The *VEGF* regulation appears to be extremely complex, with control and translational levels depending on growth factors, cytokines, lipopolysaccharides, hormones, and hypoxia [17].

Several *VEGF* region promoter polymorphisms have been linked to increased susceptibility to multiple angiogenesis-dependent diseases [16]. It is known that VEGF-mediated signaling pathways were identified that regulate angiogenesis by influencing endothelial proliferation, migration, survival, extracellular matrix degradation, and vascular/cell permeability [6,18]. Moreover, VEGF also plays an important role during the regulation of capillary actions and basal membrane permeability of the mucosa, leading to extensive edema and polyp growth [5].

The *VEGF* -141 A > C (rs28357093) SNV involves an A-to-C change and includes A and C alleles; this transition influences the variation in the circling levels of VEGF, resulting in an excessive vascular permeability [19]. This rs28357093 promotes the formation of an enzyme with modified activity that influences the *VEGF* expression, resulting in increased serum levels for the AC genotype [20]. Probably, this leads to an increase in permeability capacity, due to the upregulation for angiogenesis.

Additionally, when considering rs28357093 by different inheritance models (codominant, dominant, recessive, and over dominant), our findings describe that the mutant allele (C) may offer a risk to the development of CRSwNP. However, these findings revealed no significant association (*p* < 0.05). On the other hand, it should be noted that the number of samples was small for this pilot study. Therefore, the presence of this genetic variant must be investigated to better understand the increase in vascular permeability and expression in the inflamed nasal mucosa for both heterozygous and mutant genotypes.

Overall, our findings demonstrated that allergic rhinitis individuals’ carriers of the AC genotype present a 4-fold increase in CRSwNP risk. This evidence shows that the increased vascular permeability probably causes an inflamed nasal area. VEGF is one of the most powerful angiogenic factors, directly affecting endothelial cell proliferation, migration, and tube formation under pathological conditions. Thus, VEGF also plays an important role during the regulation of capillary actions and basement membrane permeability of nasal polyps, leading to extensive edema and polyp growth [5,16,17].

Polymorphisms in regulatory regions can affect gene expression and protein synthesis. On the other hand, the role of genetic variants from the *VEGF-A* gene in patients with CRSwNP is not clear. This outcome could help propose a hypothesis that is associated with a strong risk of developing the disease, due to altered gene transcription and protein activity levels. Moreover, new studies are necessary to assess the role of rs28357093 and other genetic variants in the susceptibility to CRSwNP in patients with allergic rhinitis, and new approaches for precision medicine.

Additionally, the high prevalence of comorbid allergies featuring an increased local production of IgE against common aeroallergens, such as salicaceae, bacterial toxins, or both, adds to the clinical complexity of various CRSwNP manifestations [21,22]. Inflammatory cells produce free radicals during phagocytosis, which is the main source of ROS [7,12,15]. Vascular endothelial dysfunction leads to oxidative stress and inflammation of vessel walls, which in turn enhances vascular endothelial dysfunction [23]. Thus, oxidative stress is associated with ROS, and this process could be associated with a possible factor in disease development [8]. The enzymatic activity of the GST system is essential for cellular protection against a range of potentially harmful agents. The absence of these enzymes can alter normal cellular functions, increasing their susceptibility to oxidative stress [11].

Nasal polys, a chronic and complicated inflammatory disorder with the formation of mostly benign polyps within bilateral nasal cavities, encompasses a series of pathological features, such as epithelial cell proliferation, eosinophil infiltration, and glandular changes [21,24]. Interestingly, chronic rhinosinusitis immune responses were shown to vary across different geographic areas and ethnicities, further contributing to disease heterogeneity. In line with this, pronounced eosinophilia with type-2-based immune responses are consistently reported in Caucasian patients with CRSwNP from Western countries, whereas non-eosinophilic CRSwNP with a type 1 and 3 immune profile is commonly demonstrated in their Asian counterparts [21].

On the other hand, considering the analysis of the dominant inheritance model, the findings described a protective effect for the *GSTT1* present genotype to CRSwNP. The GST family promotes antioxidant defenses, favoring the control of oxidative stress with a decreased production of free radicals [12]. The GST protein is responsible for combining compounds that cause oxidative stress with glutathione (GSH), a crucial part of the intracellular defense system, which protects the epithelium against the injuries and inflammation caused by oxidation [20,22]. It is probable that due to the action of GSTs in cellular detoxification, individuals with the *GSTT1* present genotype are favored regarding antioxidant defense and present a low risk to CRwNP. Dar et al. 2017 [25] found that the *GSTT1* null genotype presented a higher risk for the development of asthma. The authors considered this finding as a possible biomarker candidate for the development of allergies beside family history. Asthma is associated with nasal allergy-promoting allergic rhinitis.

Additional studies are needed to elucidate the molecular mechanisms underlying these genetic variants, and pathophysiological and genetic factors, which could contribute to these observations. Another limitation of this pilot study was that the VEGF and GST enzyme activity had not been measured. However, regarding ethnicity, our data encompassed self-declared white and non-white individuals. It is important to highlight that the Brazilian population is very diverse, with various ethnic backgrounds, a considerable number of individuals from a mixed ethnic background, and differences regarding environmental exposure and economic status. The data present ethnical heterogeneity due to the admixture of races in this population.

The Brazilian population has a great genetic diversity due to the three main ancestral contributions (Native Americans, Europeans, and Africans), and the high number of individuals from a mixed ethnic background. Moreover, Brazil is a wide territory, where different regions have different colonizers, which is reflected in the variance of genetic backgrounds of the population. This results in a large variability of skin pigmentation, which promotes a categorization of ethnicity not based on ancestry, but on the physical features of the individual, as, in Brazil, ethnic stratification is phenotypically characterized by skin color [26]. Moreover, genetic variation promotes diversity in morphological and disease-related phenotypes. Thus, this case–control study described an assessment of two genetic variants (*VEGF-A* and *GSTM1*/*GSTT1* deletion polymorphism) in the Brazilian patients to predict the CRwNP risk.

## 5. Conclusions

The findings obtained in this study suggest that Brazilian patients with allergic rhinitis carriers AC genotype (rs28357093) are more pronouncedly susceptible for CRSwNP development. This genetic variant was not yet identified as a possible susceptibility factor. However, the *GSTT1* present genotype demonstrated a protective effect favoring the antioxidant defense and allowing a low CRwNP risk. Moreover, additional studies that have larger groups in different geographic localizations may be useful to assess the association between SNVs and CRSwNP.

## Figures and Tables

**Table 1:** Characteristics of the groups with and without CRSwNP.

CRSwNP *n* (%)			
Variables	Case	Control	*p*
Age (Mean ± SD)	57.7 ± 9.9	60.47 ± 9.8	0.007 ^a^
Gender			
Female	30 (49.2)	45 (45)	0.626 ^b^
Male	31 (50.8)	55 (55)	
Smoking			
No	60 (98.4)	65 (65)	0.00000010 ^b^
Yes	1 (1.6)	35 (35)	

^a^ Student’s t test; ^b^ Fisher’s exact test; SD = Standard deviation.

**Table 2:** Genotypic and allelic frequencies of the investigated polymorphisms and their association between patients with CRSwNP and control group, using inheritance models.

Gene	Model	Genotype	Case *n* (%)	Control *n* (%)	OR (95% CI)	*p*
*rs28357093 VEGF-A*	Codominant	AA	43	77	Ref.	
		AC	14	20	1.25 (0.57–2.77)	0.56
		CC	4	3	2.38 (0.51–11.16)	0.26
	Dominant	AA	43	61	Ref.	
		AC + CC	18	23	2.26 (0.49–10.50)	0.77
	Recessive	AA + AC	57	97	Ref	
		CC	4	3	1.11 (0.53–2.30)	0.29
	Over dominant	AA + CC	47	80	Ref.	
		AC	14	20	1.19 (0.55–2.57)	0.65
Alleles		A	100	174	Ref.	
		C	22	26	1.47 (0.79–2.73)	0.22
*GSTM1*	Dominant	Present	48	65	Ref.	
		Null	13	35	0.50 (0.24–1.05)	0.06
*GSTT1*	Dominant	Present	34	71	Ref.	
		Null	27	29	0.51 (0.26–0.99)	0.05 *

* *p* ≤ 0.05.

**Table 3:** Association between *VEGF-A* and *GSTM1/GSTT1* genotypes and CRSwNP risk according to asthma, allergic rhinitis, and surgery.

**Asthma**	**Yes**	**No**			
	***n*** **(%)**	***n*** **(%)**	**OR**	**95% CI**	***p***
*VEGF-A*					
AA	26	17	Ref.		
AC	11	3	2.40	(0.58–9.87)	
CC	2	2	0.65	(0.08–5.09)	0.68
*GSTM1*					
Present	17	29	Ref.		
Null	8	7	1.94	(0.60–0.26)	0.26
*GSTT1*					
Present	19	22	Ref		
Null	6	14	0.49	(0.15–1.54)	0.22
**Allergic Rhinitis**	**Yes**	**No**			
	***n*** **(%)**	***n*** **(%)**	**OR**	**95% CI**	***p***
*VEGF-A*					
AA	11	27	Ref.		
AC	12	7	4.20	(1.31–13.50)	0.015 *
CC	1	3	0.81	(0.07–8.74)	0.86
*GSTM1*					
Present	14	23	Ref.		
Null	11	13	1.39	(0.490–3.941)	0.53
*GSTT1*					
Present	7	17	Ref.		
Null	13	24	1.31	(0.43–3.98)	0.62
**Surgery**	**Yes**	**No**			
	***n*** **(%)**	***n*** **(%)**	**OR**	**95% CI**	***p***
*VEGF-A*					
AA	13	29	Ref.		
AC	1	14	1.69	(0.50–5.71)	0.39
CC	1	3	0.15	(0.01–1.34)	0.80
*GSTM1*					
Present	9	33	Ref.		
Null	6	13	1.69	(0.50–5.71)	0.39
*GSTT1*					
Present	29	11	Ref.		
Null	17	4	1.61	(0.44–5.86)	0.46

* *p* ≤ 0.05.

## Data Availability

Not applicable.
